# High Fat Mixed Meal Tolerance Test Leads to Suppression of Osteocalcin Decrease in Obese Insulin Resistant Subjects Compared to Healthy Adults

**DOI:** 10.3390/nu10111611

**Published:** 2018-11-01

**Authors:** Urszula Razny, Joanna Goralska, Anna Zdzienicka, Anna Gruca, Barbara Zapala, Agnieszka Micek, Aldona Dembinska-Kiec, Bogdan Solnica, Malgorzata Malczewska-Malec

**Affiliations:** 1 Department of Clinical Biochemistry, Jagiellonian University Medical College, Kopernika 15a, 31-501 Krakow, Poland; joanna.goralska@uj.edu.pl (J.G.); anna.zdzienicka@uj.edu.pl (A.Z.); anna.gruca@uj.edu.pl (A.G.); barbara.zapala@uj.edu.pl (B.Z.); mbkiec@cyf-kr.edu.pl (A.D.-K.); bogdan.solnica@uj.edu.pl (B.S.); mbmalec@cyf-kr.edu.pl (M.M.-M.); 2 Department of Nursing Management and Epidemiology Nursing, Institute of Nursing and Midwifery, Jagiellonian University Medical College, Kopernika 25, 31-501 Krakow, Poland; agnieszka.micek@uj.edu.pl

**Keywords:** Bone formation in obesity, insulin resistance, osteocalcin, mixed meal tolerance test, postprandial bone turnover

## Abstract

Nutrients influence bone turnover. Carboxylated osteocalcin (Gla-OC) participates in bone formation whereas its undercarboxylated form (Glu-OC) acts as a hormone in glucose metabolism. The aim of the study was to determine the responses of Gla-OC, Glu-OC, and total-OC (calculated as the sum of Gla-OC and Glu-OC) to a high fat mixed meal tolerance test (HFMTT) in non-obese (body mass index (BMI) < 30 kg/m^2^, *n* = 24) and obese subjects (30 < BMI < 40 kg/m^2^, *n* = 70) (both sexes, aged 25–65 years). Serum Gla-OC and Glu-OC were measured at baseline as well as at 2 and 6 h during a HFMTT by enzyme-linked immunosorbent assay (ELISA). Baseline Gla-OC, Glu-OC, and total-OC levels were lower in obese individuals compared to non-obese participants (*p* = 0.037, *p* = 0.016 and *p* = 0.005, respectively). The decrease in Gla-OC and total-OC, but not in Glu-OC, concentrations during the HFMTT was suppressed in obese, but not in non-obese controls (*p* < 0.05, *p* < 0.01, *p* = 0.08, respectively). Subjects with the highest homeostatic model assessment for insulin resistance (HOMA-IR) index values had a less pronounced decrease in total-OC compared to patients with values of HOMA-IR index in the 1st quartile (*p* < 0.05). Net incremental area under Gla-OC inversely correlated with adiponectin (rho = −0.35, *p* = 0.001). Increase in insulin sensitivity and adiponectin level in obese subjects could beneficially influence postprandial bone turnover expressed by osteocalcin concentration.

## 1. Introduction

Recent clinical and epidemiologic studies have shown that obesity did not protect against osteoporosis and even could be a risk factor for fragility fractures. In fact, it was reported that in obesity, the cross-talk between adipose tissue and bone could be affected by disturbances in secretion of several adipokines and bone-derived molecules regulating bone turnover and adipogenesis as well as body weight and glucose metabolism [1,2,3,4].

Osteocalcin (OC) is a secretory product of osteoblasts, which participates not only in bone remodeling, but also in regulating energy metabolism [5,6,7,8,9]. OC undergoes vitamin-K-dependent carboxylation (Gla-OC) and interacts with hydroxyapatite crystals of the bone matrix [5,6,7]. Approximately 15% of Gla-OC is not absorbed in bones during bone formation and could be detected in the circulation. However, a portion of osteocalcin in blood remains undercarboxylated (Glu-OC) and is considered as a mediator in glucose and lipid metabolism [9,10,11]. Mice with the knockout of the osteocalcin gene (Ocn-/-) were abnormally fat and had increased adipocyte numbers as well as serum triglyceride levels. Injections of osteocalcin to these mice resulted in decreased fat mass, their triglyceride level in serum, and downregulation of lipolysis inducing genes (*Tgl* and *perilipin*) in fat [10]. Mice with deletion of the Ocn gene also presented suppression of insulin secretion, glucose tolerance, and insulin sensitivity. Analysis of their pancreas revealed reduced islets number, β-cell area, β-cell mass, and insulin content [11].

In turn, disturbances in glucose metabolism affect bone remodeling and osteocalcin secretion, which could explain increased fracture risk in subjects with diabetes mellitus type 2 (T2DM) [12,13,14]. Namely, in obese subjects with prediabetes, decreased Glu-OC blood levels were noticed, which was inversely associated with homeostatic model assessment for insulin resistance (HOMA-IR index) and insulin [15]. However, in obesity, bone metabolism could be affected not only by glucose intolerance, but also by inflammatory markers and adipokines. It has been documented that in obese subjects with low grade inflammation, Gla-OC levels were reduced in comparison to healthy volunteers and inversely correlated with the inflammatory markers, high-sensitivity C-reactive protein (hsCRP) and adipokine visfatin [15].

Among factors that could influence fat-bone interplay by regulating adipogenesis and osteoblastogenesis are caloric intake and type of nutrients [16,17,18]. It was reported that different dietary factors could lead to acute changes in bone turnover. Namely, it was documented that calcium, glucose, fat, or protein ingestion led to suppression of bone resorption of up to 50% from baseline within hours of nutrient ingestion [16,17,18,19]. However, markers of bone formation, such as procollagen type 1 amino-terminal propeptide (P1NP) and osteocalcin, are less suppressed by food intake [20,21,22].

Studies evaluating the influence of food intake on bone turnover have focused on the effect of breakfast, protein, fat, and, in particular, glucose on serum total-OC [16,17,18,19,20,21,22]. In healthy volunteers, especially after glucose overload, a significant decrease for osteocalcin (OC) was observed whereas in obese volunteers, the decrease was suppressed [20,21,22]. However, how nutrients affect bone status, especially in obesity, is not fully known. None of the previous studies explored the effect of a high fat mixed meal on serum carboxylated (Gla-OC) and undercarboxylated (Glu-OC) osteocalcins in obese subjects with insulin resistance, but without type 2 diabetes.

The aim of our study was to investigate whether carboxylated, undercarboxylated, and total osteocalcin react differently to a high fat mixed meal in patients with obesity and insulin resistance than in healthy individuals. We hypothesized that insulin resistance, or the associated inflammatory markers and adipokine disturbances in obesity, could influence OC release from bones in response to a high fat mixed meal.

## 2. Materials and Methods

### 2.1. Subjects

The study was performed in accordance with the Code of Ethics of the World Medical Association (Declaration of Helsinki) and with the Good Clinical Practice guidelines. The project was approved by the Bioethics Committee of the Jagiellonian University in Cracow, Poland (written consent, decision number KBET/82/B/2009). All participants gave written informed consent prior to participation in the study. The recruitment of study participants began in September 2009 and was completed in July 2013. Volunteers were recruited from patients at the Out-patient Clinic of Obesity and Lipid Disorder Treatment at the Department of Biochemistry, Jagiellonian University Medical College in Cracow, Poland. Obese (body mass index (BMI) 30–40 kg/m^2^) and non-obese (BMI < 30 kg/m^2^) women and men, aged 25 to 65 years were included into the study. The exclusion criteria included conditions that might affect the metabolic parameters and response to diet, such as: Chronic diseases (cardiovascular diseases, cancer, chronic inflammation), diabetes mellitus and other metabolic disorders, and kidney or liver failure. Of the 195 participants recruited for the study, 38 declined to participate and 63 subjects did not meet the inclusion criteria. Thus, a high fat mixed meal tolerance test (HFMTT) was performed in 94 participants: 70 obese volunteers (79% female, age 25–65 years) and 24 non-obese controls (71% female, age 25–65 years).

### 2.2. Anthropometric Parameters

Body mass index (BMI) was calculated by the division of body weight (kg) by height squared (m^2^). Waist to hip ratio index (WHR) was calculated by dividing the waist circumference (cm) by the measurement of the hips (cm). Circumference of waist was measured just above the belly button and the hip circumference at its widest part of the buttocks. Adipose tissue mass percentage was estimated with the bioelectrical impedance method using the Segmental Body Composition Analyser (Tanita, Japan).

### 2.3. High Fat Mixed Meal Tolerance Test (HFMTT)

Participants underwent a 6-h oral high fat mixed meal tolerance test (HFMTT), which was modified Coudrec study [23]. All patients were asked not to drink alcohol or drinks containing caffeine for 3 days before the test. The day before the HFMTT, the participants ate a low-fat meal before 6 p.m. (2 slices of bread without any fatty products and unsweetened tea). Only water could be drunk thereafter. Test breakfasts were given at 7:30 a.m., and postprandial studies were performed from 7:30 a.m. to 1:30 p.m. The composition of the HFMTT is presented in Table 1.

### 2.4. Biochemical Analyses

Venous blood samples were taken 3 times: Before the meal (fasting sample), and 2 and 6 h after breakfast (postprandial). Blood was sampled without stasis through an indwelling catheter into syringes. Within 30 min, blood samples for all measurements (except nonesterified fatty acids, NEFAs) were centrifuged at 1000× *g* for 10 min at 4 °C, and serum samples were immediately stored at −80 °C until analysis. The following biochemical analyses were performed in serum from blood samples collected before the meal: Cholesterol (total cholesterol, high-density lipoprotein (HDL) cholesterol), adipokines (leptin, adiponectin, resistin, visfatin), adhesion molecules (sE-Selectin, monocyte chemoattractant protein 1(MCP-1), soluble vascular cell adhesion protein 1 (sVCAM-1)), and proinflammatory markers (C–reactive protein (CRP), interleukin 6 (IL-6)). Total cholesterol and HDL cholesterol were measured by automated, enzymatic colorimetric methods (Allmed, Poznan, Poland) using the MaxMat Analyzer. The intra- and interassay variability coefficients were as follows: 1.4% and 3.8% (total cholesterol), 2.1% and 2.8% (HDL cholesterol), respectively. Low density lipoprotein (LDL) cholesterol was calculated according to the Friedewald formula. Serum leptin, adiponectin (adipocyte complement-related protein of 30 kDa, Acrp 30), resistin, IL-6, sE-Selectin, monocyte chemoatractant protein 1 (MCP-1), and soluble vascular cell adhesion molecule-1 (sVCAM-1) were assayed using ELISA (R&D Systems Europe, Ltd., Abingdon, UK). Within- and between-run imprecision CVs were 3% and 4% (leptin), 4% and 6% (adiponectin), 5.3% and 8.2% (resistin), 6% and 7% (IL-6), 6% and 8% (sE-Selectin), 5% and 6% (MCP-1), and 3.5% and 7.7% (sVCAM-1).

In three time points of HFMTT, the following analytes were assayed: Glucose, insulin, NEFAs, triglycerides (TGs), Gla-OC, and Glu-OC. Serum glucose and TGs were assayed by automated, enzymatic colorimetric methods (Allmed, Poznan, Poland) using the MaxMat Analyzer. The intra- and interassay variability coefficients were as follows: 2.3% and 3.5% (glucose); 1.4% and 3.4% (TGs). Serum insulin was measured by the immunoradiometric method (DIAsource, ImmunoAssays, Louvain-la-Neuve, Belgium) and read using the gamma counter (LKB Instruments, Victoria, Australia). Within- and between-run imprecision CVs were 2.1% and 6.5%, respectively. Basal insulin resistance was estimated using the homeostatic model assessment for insulin resistance (HOMA-IR index) [24]. Serum Gla-OC and Glu-OC were determined by ELISA (Takara, Tokyo, Japan). Intra- and interassay coefficients of variation were: <4.8% and <2.4% (Gla-OC), and <6.66%, and <9.87% (Glu-OC), respectively. Total osteocalcin level was calculated as the sum of Gla-OC and Glu-OC. NEFAs concentration was measured immediately in non-frozen plasma by the enzymatic quantitative colorimetric method (Roche Diagnostics GmbH, Berlin, Germany).

### 2.5. Statistical Analyses

Nominal data were analysed by chi-square (*χ*^2^) test. The Shapiro-Wilk test was used to analyse data for a Gaussian distribution and Levene’s test was used to verify the homogeneity of variance. Continuous variables were transformed if required. Normally distributed data are presented as mean ± standard error of the mean (SEM) or otherwise as median and lower-upper quartile range (25–75%). Differences between the two studied groups (non-obese vs obese) were analysed by an unpaired t test or Mann Whitney *U*-test (for non-normally distributed data). Potential differences in serum glucose, insulin, NEFA, triglycerides, and osteocalcins were calculated with repeated measures by analysis of variance -ANOVA, *p*-values are shown as follows: For differences over participants’ groups (A) or between time points (B) and for the interaction between group and time (AB). Values at single time points were compared by the Tukey post hoc test. Net incrementalareaunder HFMTT curves (Net area under curve (AUC)) included all incremental areas below the curve (below the fasting concentration) and was calculated for osteocalcins (Gla-OC, Glu-OC, total-OC) by applying the trapezoidal rule to both positive and negative osteocalcin increments and subtracting the area below the fasting level from that above [25]. Potential differences in the variation of values of Net AUC (for Gla-OC or total-OC) with quartile of biochemical parameters (HOMA-IR index or adiponectin) were analysed with the Kruskal-Wallis and Dunn test. The Spearman rank correlation was used to find an association between non-normally distributed data, *p*-values less than 0.05 were considered significant. Statistical analyses were performed with the Statistica software (StatSoft, 12 PL, Krakow, Poland).

## 3. Results

Both groups of subjects did not differ in terms of age and sex (Table 2). Although fasting blood glucose levels were similar in the studied groups of subjects, serum concentration of fasting insulin and insulin resistance index, HOMA-IR, were significantly higher (*p* < 0.001, *p* < 0.001, respectively) in obese participants compared with non-obese controls. Regarding the lipidogram, both groups of subjects had similar serum concentrations of triglycerides, LDL cholesterol, HDL cholesterol, and total cholesterol (Table 2). However, in comparison to the controls, obese subjects presented higher NEFAs concentrations in plasma (*p* = 0.048). We observed that higher adipose tissue mass (*p* < 0.001) probably leads to increased serum concentrations of adipokines: Leptin (*p* = 0.001) and resistin (*p* = 0.007); as well as higher blood levels of proinflammatory markers: Il-6 (*p* = 0.010) and hsCRP (*p* = 0.008) in obese subjects in comparison to non-obese controls. However, blood concentration of other adipokines (adiponectin, visfatin) and MCP-1 as well as cell adhesion molecules (sE-selectin, sVCAM-1) did not differ between the studied groups of subjects. As expected, serum osteocalcins’ concentration was lower in obese than non-obese participants: Gla-OC (*p* = 0.037), Glu-OC (*p* = 0.016), and total-OC (*p* = 0.005).

**Figure 1:**
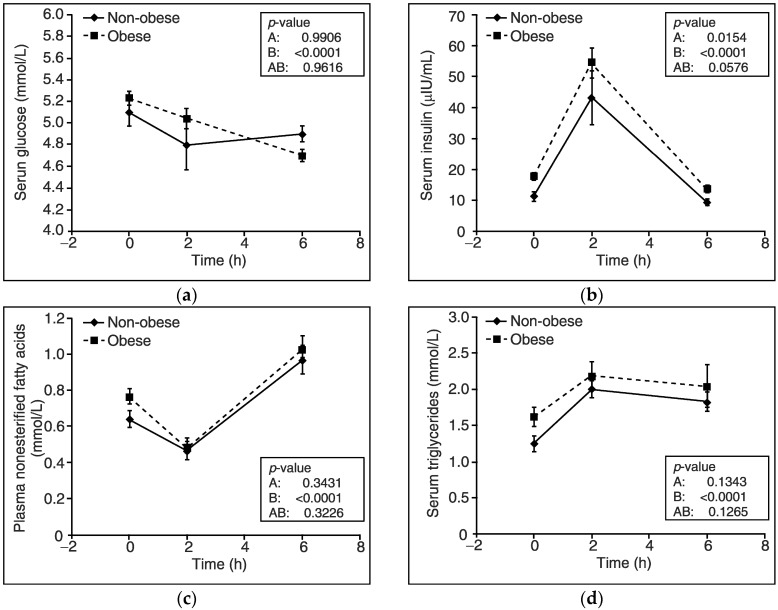
Changes in blood levels of glucose, insulin, nonesterified fatty acids, and triglycerides during a high fat mixed meal tolerance test in obese (*n* = 70) and non-obese subjects (*n* = 24). (**a**) Glucose concentration in serum. (**b**) Insulin concentration in serum. (**c**) Nonesterified fatty acids concentration in plasma. (**d**) Triglycerides concentration in serum. Values are presented as means ± SEM. Statistics: Analyses were performed with repeated-measures by ANOVA and denote differences between non-obese and obese participants (A), differences over time (B), and differences owing to an interaction between group and time (AB).

When analyzing the response to the HFMTT, we found that plasma levels of glucose were gradually lowered after 2 (by about 5%) and 6 (by approximately 10%) hours of the test (*p* < 0.0001) without any differences between the studied groups of subjects (Figure 1a). In turn, insulin values during HFMTT reached a maximum after 2 h of the test, then were gradually reduced (*p* < 0.0001), and were higher for obese than non-obese participants (*p* = 0.015) (Figure 1b). Although obese subjects presented higher plasma baseline levels of NEFAs than non-obese participants, we did not observe any differences in response to the HFMTT between the studied groups of subjects (Figure 1c). Namely, in both groups of participants, plasma levels of NEFAs reached a minimum after 2 h of the test and afterwards gradually increased above baseline values after 6 h (*p* < 0.0001). Regarding the TGs serum level during the HFMTT, it was similar in obese and non-obese subjects. Serum TGs’ concentration was increased after 2 h and gradually reduced after 6 h of the test in both groups of patients (*p* < 0.0001) (Figure 1d).

**Figure 2:**
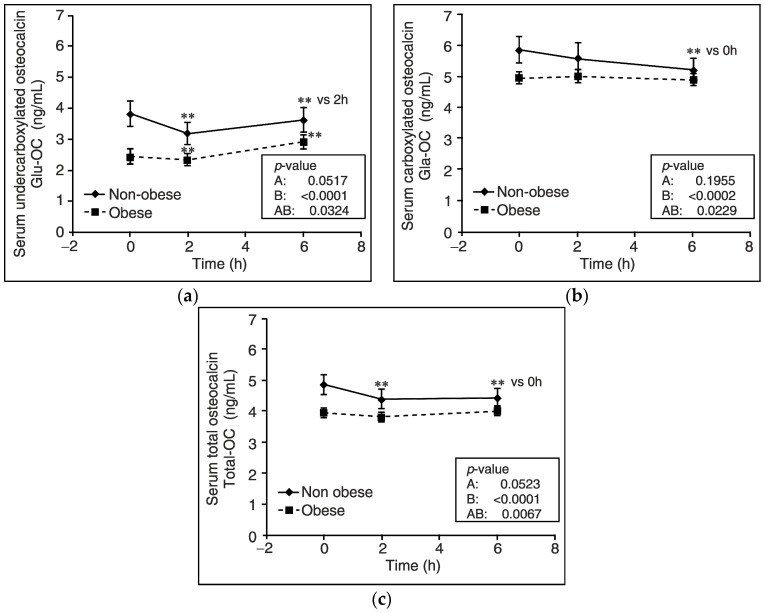
Changes in blood levels of osteocalcins during a high fat mixed meal tolerance test in obese (*n* = 70) and non-obese subjects (*n* = 24). (**a**) Undercarboxylated osteocalcin (Glu-OC) concentration in serum. (**b**) Carboxylated osteocalcin (Gla-OC) concentration in serum. (**c**) Total osteocalcin (total-OC) concentration in serum. Values are presented as means ± SEM. Statistics: Analyses were performed with repeated-measures by ANOVA and denote differences between non-obese and obese participants (A), differences over time (B), and differences owing to an interaction between group and time (AB). Values at single time points were compared by the Tukey post hoc test. Significant differences are indicated as follows: ** *p* < 0.01.

Among osteocalcin levels, we observed that Gla-OC levels in serum during HFMTT differed between non-obese and obese participants (*p* = 0.0229) (Figure 2b). In non-obese subjects, after 6 h, but not after 2 h, of the HFMTT, Gla-OC concentration was significantly reduced (by about 12%) in comparison to baseline values (*p* < 0.01). This effect was suppressed in obese participants (Figure 2b).

**Figure 3:**
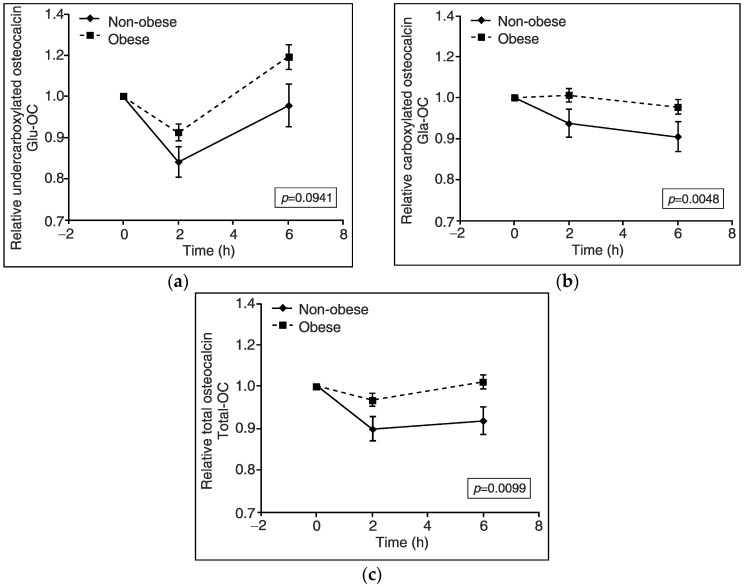
Relative changes in blood levels of osteocalcins during a high fat mixed meal tolerance test in obese (*n* = 70) and non-obese subjects (*n* = 24). (**a**) Relative undercarboxylated osteocalcin (Glu-OC) concentration in serum. (**b**) Relative carboxylated osteocalcin (Gla-OC) concentration in serum. (**c**) Relative total osteocalcin (total-OC) concentration in serum. Values are presented as means ± SEM. Statistics: The total areas under curves (AUCs) were measured by the trapezoidal method and differed between non-obese and obese subjects in Gla-OC (*p* = 0.0048, unpaired *t*-test) and total-OC (*p* = 0.0099, unpaired *t*-test), but not in Glu-OC (*p* = 0.0941, Mann-Whitney *U*-test).

**Figure 4:**
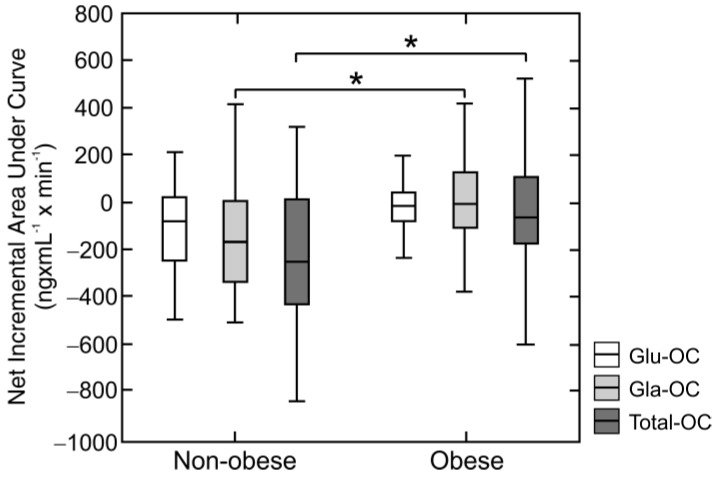
Net incremental areas under blood osteocalcins levels during a high fat mixed meal tolerance test in obese (*n* = 70) and non-obese subjects (*n* = 24). Net incremental areas (Net AUC) under undercarboxylated (Glu-OC), carboxylated (Gla-OC), and total osteocalcins during a high fat mixed meal tolerance test were calculated by the trapezoidal rule to both positive and negative osteocalcins increments and subtracting the area below the fasting level from that above. Values are presented as medians (line), upper and lower quartile (box), and extremes (whiskers). Statistics: Mann-Whitney U-test denotes differences between the non-obese and obese participants. Significant differences are indicated as follows: * *p* < 0.05.

Moreover, relative Gla-OC AUC value (*p* = 0.005) and absolute value of the net incremental Gla-OC AUC were lower in obese subjects than non-obese controls (*p* < 0.05) (Figure 3b and Figure 4). We also found that total-OC plasma concentration was gradually reduced after 2 and 6 h of the HFMTT in non-obese subjects (*p* < 0.01), but not in obese volunteers (Figure 2c), which was reflected by values of the relative total-OC AUC as well as the absolute value of the net incremental total OC AUC, which were lower in obese than non-obese subjects (*p* = 0.036, *p* = 0.01, *p* < 0.01) (Figure 3c and Figure 4). Unexpectedly, serum concentrations of Glu-OC during the HFMTT were similar in non-obese and obese subjects. Namely, serum Glu-OC levels were decreased after 2 h of the HFMTT (*p* < 0.001) and increased after 6 h of the test, (*p* < 0.001) (Figure 2a). There was a tendency for a lower value of relative Glu-OC AUC (*p* = 0.09) in obese subjects compared with non-obese controls (Figure 3a).

Analyses of correlations (Table 3) revealed that BMI was positively associated with the net incremental area under total-OC (rho = 0.28, *p* = 0.007) and Gla-OC osteocalcin (rho = 0.25, *p* = 0.014). Net incremental total OC AUC was also inversely correlated with adiponectin (rho = −0.27, *p* = 0.011) and positively associated with fasting insulin (rho = 0.30, *p* = 0.007) and HOMA-IR (rho = 0.28, *p* = 0.008).

**Figure 5:**
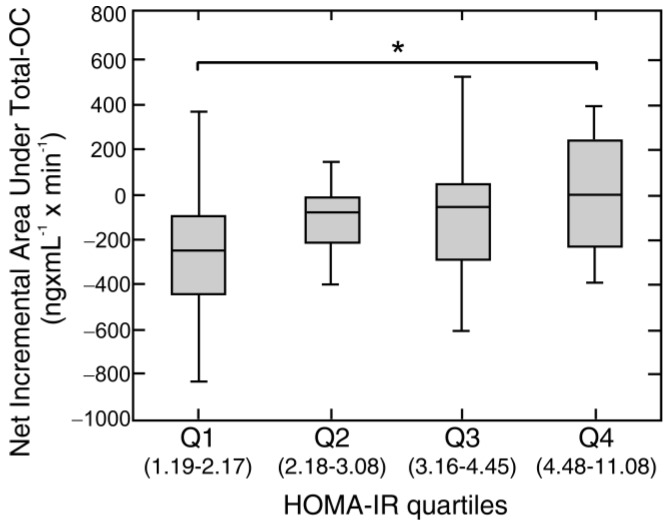
Net incremental areas under total-OC and quartiles of the Homeostatic Model Assessment of Insulin Resistance (HOMA-IR) index. Variation of values of net incremental areas (Net AUC) under total osteocalcin (Total-OC) during a high fat mixed meal tolerance test with the quartile of the HOMA-IR (range of values for each quartile is in brackets) in combined non-obese (*n* = 24) and obese (*n* = 70) subject study groups. Values are presented as medians (line), upper and lower quartile (box), and extremes (whiskers). Statistics: Kruskal-Wallis and Dunn test. Significant differences are indicated as follows: * *p* < 0.05.

With the strong positive correlation of HOMA-IR with the net incremental total OC AUC, the subjects were classified by HOMA-IR quartiles and net incremental total OC AUCs were compared between these groups. In quartile 4 of HOMA-IR, there was a lower absolute value of the net incremental total OC AUC with respect to quartile 1 (*p* < 0.05) (Figure 5).

**Figure 6:**
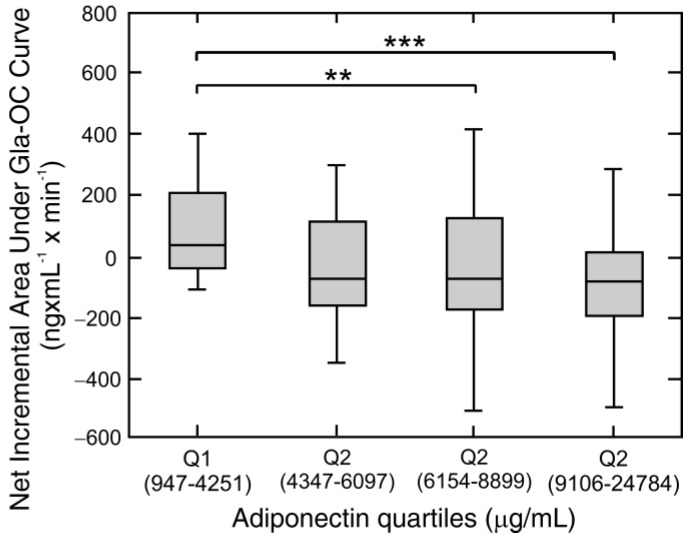
Net incremental areas under Gla-OC and quartiles of serum adiponectin concentration. Variation of values of net incremental areas (Net AUC) under carboxylated osteocalcin (Gla-OC) during a high fat mixed meal tolerance test with the quartile of adiponectin serum concentration (range of values for each quartile is in brackets) in combined non-obese (*n* = 24) and obese (*n* = 70) subject study groups. Values are presented as medians (line), upper and lower quartile (box), and extremes (whiskers). Statistics: Kruskal-Wallis and Dunn test. Significant differences are indicated as follows: ** *p* < 0.01 for difference in values of Gla-OC Net AUC between quartile 1 and quartile 3 of serum adiponectin concentration. *** *p* < 0.001 for difference in values of Gla-OC Net AUC between quartile 1 and quartile 4 of serum adiponectin concentration.

We also found that the net incremental Gla-OC AUC was positively associated with fasting insulin (rho = 0.22, *p* = 0.042) and inversely correlated with adiponectin levels (rho = −0.34, *p* = 0.001). With the strong negative correlation of net incremental Gla-OC AUC and adiponectin, subjects were re-classified by adiponectin quartiles and net incremental Gla-OC AUC levels were compared between quartiles. Absolute values of net incremental Gla-OC AUC were higher in quartile 3 and quartile 4 of adiponectin level with respect to quartile 1 (*p* < 0.01, *p* < 0.001) (Figure 6).

## 4. Discussion

Our studies are the first to show the effect of a high fat mixed meal on the blood level of not only total osteocalcin, but also its carboxylated and undercarboxylated form, in obese insulin resistant subjects. Studies performed so far have shown that different nutrients could influence bone turnover [16,17,18,19,20,21,22]. However, as a marker of bone formation, blood total osteocalcin has been used. In our studies, we focused on the effect of more physiological intake of nutrients during a high fat mixed meal on the blood levels of the carboxylated and undercarboxylated form of osteocalcin in non-obese healthy individuals as well as in insulin resistant obese volunteers. We observed a suppression of total osteocalcin (the sum of carboxylated and undercarboxylated osteocalcin) serum levels after 2 and 6 h of mixed meal consumption in healthy volunteers. Our results appear to agree with previous studies [26] Namely, Henriksen et al. indicated that fat (35 g) and protein (35 g) present a tendency to suppress total blood osteocalcin concentration during 3 h of food consumption. In our study, we observed a statistically significant decrease of total osteocalcin, which could be associated with the fact that our breakfast consisted of three combined nutrients (protein, fat, and saccharides) and its fat content was approximately 2.5 times higher (83.7 g vs 35 g) than in the study reported by Henriksen, whereas protein content was comparable [26]. Regarding the suppression of total serum osteocalcin levels after 6 h of the HFMTT, similar results were also noticed in studies by Henriksen, especially after protein as well as fat intake. In healthy volunteers, we also observed an approximately 10% baseline decrease of serum carboxylated osteocalcin (Gla-OC) level 6 h after breakfast consumption. Gla-OC accumulates mainly in bone (binding to the hydroxyapatite lattice), and only approximately 15% of Gla-OC is released into the bloodstream. Thus, a decrease in serum Gla-OC concentration after breakfast could suggest bone turnover in favour of bone formation to maximize skeletal strength and bone mass when nutrition is abundant in physiological conditions. Our studies have for the first time demonstrated that a decrease of total and carboxylated osteocalcin after a high fat mixed meal was suppressed in obese individuals who did not develop diabetes and also did not have any fasting and postprandial lipid disturbances. Obese subjects included in the intervention study presented insulin resistance (higher HOMA-IR index) and low grade inflammation (high serum IL-6 and hsCRP concentration) whereas baseline blood Gla-OC, Glu-OC, and total osteocalcin levels were lower than in non-obese controls. Suppression of baseline serum osteocalcin (Gla-OC and total-OC) and its postprandial decrease could indicate bone turnover disturbances in obese individuals. It has been reported that in the obese bone formation index, defined as quotient procollagen type 1, amino-terminal propeptide to bone alkaline phosphatase (PINP/BAP) was lower [21]. Moreover, osteoblasts from obese subjects were less active and produced less collagen. During the HFMTT the absolute value of net incremental AUC for Gla-OC and total-OC in obese subjects was lower than in the control group. Net incremental total-OC AUC was positively associated with BMI, fasting insulin, and HOMA-IR. BMI and fasting insulin were also positively associated with net incremental Gla-OC AUC. Thus, in our studies, the different response of obese and non-obese subjects to the HFMTT appears to be associated with disturbances in glucose metabolism. It has previously been presented that hyperglycemia could contribute to lower bone mass and a lower rate of bone formation [13,26]. Advanced glycation end products (AGEs) contributed to reduced synthesis of type I collagen and osteocalcin in osteoblast-like cells [27]. Moreover, increased AGEs and protein oxidative damage markers in obesity may contribute to decreased Gla-OC levels [28]. However, our obese subjects had normal blood glucose, but had insulin resistance and high fasting blood insulin. During the HFMTT, postprandial insulin blood concentration was higher in obese than control subjects. Fasting glucose, glucose during the HFMTT, and post-HFMTT glucose did not differ between the groups. All obese subjects were normoglycemic despite insulin resistance and obesity, which is considered a risk factor for impaired glucose tolerance [29]. Our results agree with studies performed in mice, which presented that knockout of the insulin receptor in osteoblasts resulted in decreased secretion of osteocalcin [30]. Constantly elevated insulin levels during the HFMTT in obese subjects could contribute to suppression of bone OC and Gla-OC content and, in consequence, the lack of osteocalcins’ ability to react to a food challenge. Pollock et al. [31] supported that in humans, Gla-OC may be more relevant to insulin sensitivity whereas Glu-OC could influence β-cell function. This effect of Glu-OC on β-cell function appeared to be important in subjects with prediabetes and diabetes, but not in healthy individuals [31]. We did not observe a suppression of postprandial Glu-OC decrease in obese normoglicemic subjects in comparison to non-obese participants. Schwetz et al. [22] presented postprandial suppression of Glu-OC blood decrease in obese insulin resistant subjects, but the decline was noticed after acute oral glucose load (75 g). In our studies, the high fat mixed meal contained 30.6 g saccharides and postprandial glucose level did not change dramatically in obese as well as non-obese subjects, which could explain the lack of differences in Glu-OC levels between the two studied groups of patients. In turn, Viljakinen [21] speculated that lower bone turnover in obese volunteers after glucose intake was dependent on leptin blood concentration. Leptin correlated inversely with all markers of bone turnover, especially in obese volunteers. We did not find any associations between osteocalcins and leptin. However, net incremental total-OC AUC and Gla-OC were inversely associated with adiponectin. Adiponectin is secreted predominantly by differentiated adipocytes and participates in bone metabolism. Adiponectin could promote the osteogenic differentiation of the mesenchymal stem cells (MSCs) by increasing the expression of alkaline phosphatase (BAP), osteocalcin (OC), and type I collagen (Col-I) [32]. Moreover, adiponectin stimulates the proliferation, maturation, and mineralization of osteoblasts [33,34,35]. On the other hand, it has been reported that osteocalcin can directly induce expression of adiponectin in adipose tissue [10]. It is well known that adiponectin participates in insulin sensitivity [36], however, a recent study in mice reported that this anti-inflammatory adipokine regulates bone mass in mice fed a normal diet, without affecting glucose metabolism [37]. Our studies reported that subjects with higher serum adiponectin concentrations were associated with suppressed postprandial blood concentrations of Gla-OC and total OC. Such results could suggest that the majority of osteocalcins were bound in bone. The response of bone turnover markers to feeding could be elicited by incretin hormones, such as glucagon–like peptide (GLP-2) or glucose dependent insulinotropic peptide (GIP) [38]. However, studies by Christiensen indicated that GIP infusions inhibited bone resorption markers, but had no effect on bone formation [39]. In turn, GLP-2 regulated bone turnover [19,40], but osteocalcin concentration remained unaffected by GLP-2 administration [19]. The strengths of our study include measurement of different forms of OC during a high fat mixed meal test, which is a more physiological intake of nutrients than oral glucose tolerance test (OGTT) in obese subjects in comparison to non-obese subjects. It is a novel study to investigate the relationship between bone turnover and energy metabolism in obesity. However, our study has potential limitations. Firstly, the number of subjects included in the study is small and the number of both sexes is unequal. Secondly, the number of subjects in the control group is small in comparison to obese individuals. Another limitation is the fact that we did not measure vitamin K in subjects involved in the study. However, it is unlikely that they had severe vitamin K deficiency because subjects included in the study were not on parenteral feeding and had no dietary restrictions. The overall bone remodeling is regulated by cytokines, hormone interactions, and the “circadian rhythm”. However, studies showed that the majority of the variations observed in biochemical markers of bone resorption over the day and night is not circadian, but rather induced by food intake [19]. One of the limitations of the study is also the fact that we measure one marker of bone remodeling—osteocalcin. Osteocalcin (bone gamma-carboxyglutamic acid-containing protein) is a noncollagenous protein produced by osteoblasts, and is regarded as a marker of bone formation (serum osteocalcin levels correlate with bone formation rates), but it is suggested to be involved in the process of mineralization rather than matrix production. Osteocalcin is a late marker of bone formation and its production is very low during bone development and reaches maximal levels at late stages of mineralization. Its measurements in blood has some limitations because this protein has a short half-life, the intact molecule is unstable, and its concentration depends on renal function and vitamin K status. To reflect different aspects of osteoblast function, other bone formation markers should be measured as: Byproducts of collagen synthesis (N-terminal propeptide of type I procollagen (PINP)) and bone-specific alkaline phosphatase (BSAP). To estimate bone turnover also, markers of bone resorption should be assayed in serum as C-terminal telopeptide of type I collagen (CTX). We did not measure growth hormone and cortisol in blood of our volunteers. Both groups had insufficient vitamin D status and in the obese group, vitamin D concentration was lower than in controls (data not show). It was reported that vitamin D deficiency could contribute to elevated parathormone (iPTH), leading to enhanced bone turnover. The circadian variation in serum cortisol may be partly responsible for the pattern of bone formation markers, since it has been demonstrated that infusion of cortisol in the morning depresses osteocalcin secretion during the day. However, we postulate that our results could be a basis for further studies evaluating the role of food intake on postprandial bone turnover. We also believe that our study could provide basic knowledge contributing to better understanding the influence of nutrients on obesity related bone remodeling disorders.

## 5. Conclusions

Our findings suggest that in obesity, a high fat mixed meal affects postprandial bone turnover and may contribute to bone remodeling disorders by leading to suppression of bone formation markers’ (Gla-OC, total-OC) decrease in blood. It could be suggested that in obesity, postprandial bone turnover expressed by blood osteocalcin concentration could be beneficially influenced by an increase in insulin sensitivity and blood adiponectin levels. However, further studies in larger groups of subjects are necessary to investigate the mechanism of the influence of nutrients on postprandial bone formation markers in obesity.

## Figures and Tables

**Table 1:** Composition of high fat mixed meal tolerance test (HFMTT).

Components of HFMTT	Amount (g)	Caloric Value (kcal)	Protein (g)	Carbohydrates (g)	Fat (g)	SFA (g)	MUFA (g)	PUFA (g)
Wheat bread	50	124.5	3.4	28.0	0.7	0.1	0.08	0.3
Butter	20	147.0	0.1	0.1	16.5	10.9	4.5	0.2
Cream cheese	60	178.8	8.1	0.7	16.2	9.7	5.2	0.4
Roasted pork	100	291.0	30.4	0.7	18.7	5.5	9.7	2.3
Mayonnaise	40	285.7	0.5	1.0	31.6	3.4	6.4	20.1
Sum	270	1027.0	42.5	30.6	83.7	29.6	25.8	23.4
en%			16	11	73			
g/kg diet			157.4	113.3	310.0	109.7	95.6	86.7

Abbreviations: en%, energy%; MUFA, monounasaturated fatty acids; PUFA, polyunsaturated fatty acids; SFA, saturated fattyacids.

**Table 2:** Comparison of anthropometric and biochemical parameters between non-obese and obese subjects.

Parameter	Non-Obese (*n* = 24)	Obese (*n* = 70)	*p*-Value *
Age (years)	47.52 ± 2.22 ^2^	46.38 ± 1.40	0.62
Sex, female (%)	79	71	0.46
BMI (kg/m^2^)	28.58 (27.47–29.08) ^1^	34.16 (32.13–36.28)	<0.001
WHR (waist to hip ratio)	0.85 (0.82–0.92)	0.87 (0.82–0.97)	0.59
Adipose tissue mass (%)	35.00 (32.35–38.20)	40.40 (34.48–43.10)	<0.001
Systolic BP (mm Hg)	120 (110–124)	130 (120–140)	0.019
Diastolic BP (mm Hg)	80 (70–85)	82 (80–90)	0.088
Total Cholesterol (mmol/L)	5.43 ± 0.19	5.48 ± 0.13	0.82
HDL Cholesterol (mmol/L)	1.29 ± 0.04	1.28 ± 0.03	0.83
LDL Cholesterol (mmol/L)	3.57 ± 0.17	3.51 ± 0.12	0.79
NEFA (mmol/L)	0.66 ± 0.04	0.76 ± 0.03	0.048
TG (mmol/L)	1.09 (0.81–1.41)	1.31 (0.88–2.01)	0.10
Glucose (mmol/L)	5.26 ± 0.11	5.19 ± 0.06	0.93
Insulin (µIU/mL)	10.00 (7.80–11.10)	16.45 (11.68–20.85)	<0.001
HOMA-IR	2.17 (1.92–2.57)	3.49 (2.37–4.66)	<0.001
Leptin (ng/mL)	22.64 ± 2.29	39.94 ± 2.75	0.001
Adiponectin (µg/mL)	6.76 ± 0.63	7.06 ± 0.52	0.62
IL-6 (pg/mL)	0.89 (0.73–1.30)	1.30 (0.90–1.91)	0.010
hsCRP (mg/L)	0.80 (0.38–1.82)	2.22 (0.99–3.89)	0.008
Resistin (ng/mL)	9.60 ± 0.67	10.14 ± 0.49	0.007
Visfatin (ng/mL)	1.07 ± 0.21	1.16 ± 0.09	0.39
sE-selectin (pg/mL)	34.08 ± 3.26	39.51 ± 1.68	0.09
MCP-1(pg/mL)	343.91 ± 19.41	363.06 ± 11.99	0.23
sVCAM-1 (ng/mL)	582.93 ± 24.05	622.37 ± 18.59	0.25
Glu-OC (ng/mL)	3.95 ± 0.42	2.92 ± 0.26	0.016
Gla-OC (ng/mL)	5.89 ± 0.45	4.95 ± 0.20	0.037
Total-OC (ng/mL)	9.84 ± 0.64	7.87 ± 0.32	0.005

* Significant difference between non-obese (*n* = 24) and obese group (*n* = 70) (unpaired *t*-test or Mann-Whitney *U*-test for non-normally distributed variables, nominal data were analysed by *χ*^2^ test), *p* < 0.05. ^1^ Median (25–75%); all such values. ^2^ Mean ± SEM; all such values. Abbreviations: BMI, body mass index; BP, blood pressure; Gla-OC, carboxylated osteocalcin; Glu-OC, undercarboxylated osteocalcin; HDL, high-density lipoprotein; HOMA-IR, homeostatic model assessment for insulin resistance; hsCRP, high-sensitivity C-reactive protein; NEFA, nonesterified fatty acids; LDL, low-density lipoprotein; IL-6, interleukin 6; MCP-1, monocyte chemoattractant protein 1; OC, osteocalcin; sVCAM-1, soluble vascular cell adhesion protein 1; TGs, triglycerides; WHR, waist to hip ratio.

**Table 3:** Spearman rank correlation between net incremental area (under total osteocalcin and carboxylated osteocalcin) and BMI and biochemical parameters.

Parameter	Net Incremental Total-OC AUC		Net Incremental Gla-OC AUC	
	rho	*p*	rho	*p*
BMI	0.28	0.007	0.26	0.014
Fasting Insulin	0.30	0.007	0.23	0.042
HOMA-IR	0.29	0.008	0.19	0.07
Adiponectin	−0.27	0.011	−0.35	0.001

Correlations with *p* < 0.05 were considered significant in combined non-obese (*n* = 24) and obese (*n* = 70) volunteers. Abbreviations: AUC, area under curve; BMI, body mass index; Gla-OC, carboxylated osteocalcin; HOMA-IR, Homeostatic Model Assessment of Insulin Resistance; OC, osteocalcin.
